# Nutritional Status as a Mediator of Fatigue and Its Underlying Mechanisms in Older People

**DOI:** 10.3390/nu12020444

**Published:** 2020-02-10

**Authors:** Domenico Azzolino, Beatrice Arosio, Emanuele Marzetti, Riccardo Calvani, Matteo Cesari

**Affiliations:** 1 Geriatric Unit, Fondazione IRCCS Ca’ Granda Ospedale Maggiore Policlinico, 20122 Milan, Italy; beatrice.arosio@unimi.it (B.A.);; 2 Department of Clinical Sciences and Community Health, University of Milan, 20122 Milan, Italy; 3 Fondazione Policlinico Universitario “Agostino Gemelli” IRCCS, 00168 Rome, Italy; emarzetti@live.com (E.M.); riccardo.calvani@gmail.com (R.C.); 4 Institute of Internal Medicine and Geriatrics, Università Cattolica del Sacro Cuore, 00168 Rome, Italy

**Keywords:** exhaustion, aging, nutrition, sarcopenia, frailty, aging, mitochondria, inflammation, cytokines

## Abstract

Fatigue is an often-neglected symptom but frequently complained of by older people, leading to the inability to continue functioning at a normal level of activity. Fatigue is frequently associated with disease conditions and impacts health status and quality of life. Yet, fatigue cannot generally be completely explained as a consequence of a single disease or pathogenetic mechanism. Indeed, fatigue mirrors the exhaustion of the physiological reserves of an older individual. Despite its clinical relevance, fatigue is typically underestimated by healthcare professionals, mainly because reduced stamina is considered to be an unavoidable corollary of aging. The incomplete knowledge of pathophysiological mechanisms of fatigue and the lack of a gold standard tool for its assessment contribute to the poor appreciation of fatigue in clinical practice. Inadequate nutrition is invoked as one of the mechanisms underlying fatigue. Modifications in food intake and body composition changes seem to influence the perception of fatigue, probably through the mechanisms of inflammation and/or mitochondrial dysfunction. Here, we present an overview on the mechanisms that may mediate fatigue levels in old age, with a special focus on nutrition.

## 1. Overview

Aging is accompanied by substantial changes in body composition, the most notable of which are a reduction of lean body mass and increased adipositiy. These modifications are important contributors to the onset of frailty in older persons. Frailty is a complex condition characterized by reduced homoeostatic reserves, resulting in increased vulnerability to stressors. It is associated with several adverse outcomes, including falls, hospitalization, functional decline, and mortality [1,2]. Several tools have been developed and validated over the years for identifying frail individuals. Interestingly, most of the available tools, more or less explicitly, include the concept of fatigue [1]. In other words, fatigue seems to be often considered a typical manifestation of the aging individual, a symptom making evident the effects of advancing age on the organism.

Despite its high prevalence and the burden it poses over the person, fatigue remains an often neglected symptom. It is usually defined as a state associated with the weakening and/or depletion of the individual’s physical and/or mental resources. Its heterogeneous phenotype is described as ranging from a general state of lethargy to a very specific work-induced burning muscular sensation. It leads to the inability to continue functioning at the normal level of activity [3]. Physical fatigue, although widespread in everyday life, usually becomes particularly noticeable during heavy exercise. On the other hand, mental fatigue most often manifests as somnolence [3]. Despite its inclusion in the DSM-5, fatigue is often defined and operationalized in heterogeneous ways. Depending on how the symptom is referred, several terms are interchangeably used to define fatigue, such as tiredness, exhaustion, fatigability, low vitality, anergia, weakness, and lassitude [4,5]. Tiredness, which is one of the most widely used terms to describe the symptom of fatigue, has been depicted as a transitory decrease of strength and energy [6], and can be relieved by sleep and rest [4]. On the other hand, “vital exhaustion” is a conceptualization of fatigue often used in the cardiology setting, and encompasses lack of energy, increased irritability, and the feeling of demoralization [5].

Fatigue is strongly associated with poor physical performance [7], and represents a strong predictor of multiple negative outcomes in older persons (including hospitalizations, increased use of healthcare services, incident disability, mortality) [8,9,10]. It is frequently observed in many diseases (e.g., cancer, neurodegenerative disorders, rheumatologic disease, heart failure) and can directly and indirectly affect the individual’s health status and quality of life. Indeed, fatigue is a prolonged symptom in many neurologic and muscular disorders, such as multiple sclerosis, myasthenia gravis and fibromyalgia affecting both the psychological and the physical domain [11,12]. On the other hand, fatigue may be an isolated and persistent symptom with unknown etiology, the so-called “chronic fatigue syndrome” (CFS) [5,11]. Nevertheless, for many older persons, fatigue can hardly be univocally ascribed to or completely explained by a single disease. It often remains an unexplained symptom with unclear pathophysiological mechanisms [13]. Yet, fatigue may represent the clinical alert launched by the organism with a limited homeostatic capacity in front of a disproportionate stressor challenging it [14]. 

Although the prevalence of fatigue is estimated to be between 6% and 45% in community-dwelling older persons [15], these figures are likely underestimating the phenomenon. In fact, fatigue is a largely neglected symptom in both clinical and research settings. Given the lack of a gold standard for the assessment of fatigue, its evaluation in older persons requires a complex methodological framework that needs to take into account the underlying effects of the aging process (altering the standard definitions of normality versus abnormality). Moreover, the subjective symptom of fatigue does not yet receive adequate attention in clinical settings, at least not as much as other similar/related conditions (e.g., depression and pain). Like fatigue, pain and depression are subjective feelings characterized by high prevalence older persons and are associated with negative clinical endpoints [16,17]. Fatigue, pain and depression often coexist and synergistically act in the vicious circle of the disabling process. However, if pharmacological (largely symptomatic) remedies exist for pain and depression, no effective treatments are currently available for alleviating the burdening symptom of fatigue. The most promising ways to understand the pathophysiological mechanisms underlying fatigue seem to be sleep parameters, autonomic nervous system abnormalities, and biological complexity [4,14]. In particular, nutritional status plays an important role as a mediator of fatigue both directly and indirectly by mediating its underlying mechanisms. Fatigue may, in fact, mimic the exhaustion of the metabolic reserves of the individual. In this context, the aim of the present article was to provide an overview of the patterns, with a special focus on nutrition, which may influence fatigue levels in older people. 

## 2. Biological Complexity

Fatigue is a quite complex and multidimensional symptom. The underlying mechanism potentially modulating fatigue are not completely known but may include quite a lot of biological patterns which might help understand it. Modifications of cardiac function, chronic inflammatory status, skeletal muscle modifications, nutritional deficiencies and sleep disorders may influence fatigue [4,5]. General cardiac functioning [18] and reduced aerobic capacity [9,19,20] are related to fatigue. Pain may contribute to fatigue by increasing heart rate, blood pressure, respiratory rate, muscle tone, and oxygen consumption [21]. Furthermore, sleep disorders both in qualitative and quantitative domains (which are common in the older population) may contribute to the fatigue onset. In fact, it has been reported that people with a short sleep duration are usually more fatigued than those who sleep for longer periods [22]. Noticeably, older persons who report sleeping problems are more fatigued than others [23]. Sleep deprivation has also been associated with metabolic alterations. Among healthy individuals with experimentally induced sleep-deprivation, cortisol levels and calorie intake increase, while glucose tolerance and leptin levels decrease [24,25]. Sleepiness and fatigue are difficult to differentiate. The two conditions may lead to a reduction in physical activity or induce consumption of more calories as an attempt to boost energy levels [26]. Moreover, modifications in body composition and dietary changes may contribute both to fatigue and its underlying conditions (i.e., sleep disorders, depression, skeletal muscle modifications). 

## 3. Assessment

Given the subjective nature of the symptom, the measurement of fatigue is quite complex. Indeed, despite the several tools have been validated to measure fatigue and may be predictive of adverse outcomes, none of them is considered as a gold standard [4,5]. For example, unidimensional scales are focused on specific features of the symptom (i.e., fatigue severity) and on specific populations (i.e., cancer patients). This is the case of the Brief Fatigue Inventory [27], which is a 9-item scale developed in the oncology setting, measuring fatigue severity. On the other hand, multidimensional tools are more detailed and provide information about the nature of the symptom, its intensity and the impact on activities of daily living (ADL) [5]. To date, the Multidimensional Fatigue Inventory is a 20-item scale, validated both under normal and pathologic conditions, which includes different aspects of the symptom such as its nature (i.e., general, physical or mental), reduced activities and motivation [28]. Several other tools, including the concept of fatigue, may provide information about fatigue. For example, the Short Form-36 (SF-36) Vitality scale, is a part of the Short-Form-36 Health Survey developed as a generic indicator of health status [29,30]. The SF-36 Vitality scale consists of four items based on energy and fatigue and has been validated both in general and pathologic populations. Another example is the criterion of “exhaustion” included in the frailty phenotype proposed by Fried et al. in the Cardiovascular Health Study Frailty Screening Scale [31,32]. However, this operationalization does not completely capture the multidimensionality of the symptom.

## 4. Modifications of Food Intake

Advancing age is characterized by multiple changes (e.g., physiological, psycho-social and pathological changes) (Table 1) leading to poor appetite and impacting on the quantity and/or quality of food consumed [33]. This condition has been defined as “anorexia of aging”, a disorder potentially leading to malnutrition [34,35]. 

Undernutrition may result in weight loss and nutritional deficiencies leading to fatigue by means of “lack of energy” (a key concept of fatigue) [5,36,37,38]. When protein and energy intakes fail to meet individuals need, body stores are catabolized to provide energy, leading to the depletion of body fat and muscle [39,40] with consequent symptoms such as fatigue or tiredness [36,38]. Poor nutritional status is also associated with reduced physical performance leading to fatigue [38]. Furthermore, there appears to be a bi-directional association between undernutrition and fatigue. In fact, while undernourished people are more prone to experience fatigue symptoms, fatigued people may be at risk of undernutrition by means of a lack of energy to prepare a meal [37]. 

Chronic low-grade inflammation, a hallmark of the aging process, is acknowledged as one of the major determinants of age-related anorexia. The so-called inflamm-aging is characterized by increased circulating concentrations of cytokines (e.g., interleukin (IL) 1, IL-6, and tumor necrosis factor alpha (TNF-α)) [35,41], responsible for a reduction of food intake, altered metabolism (i.e., elevation of resting energy expenditure), and increased muscle catabolism, eventually resulting in the risk of malnutrition [42]. Additionally, it is well documented that micronutrient deficiencies may determine oxidative stress and consequently, feed a vicious circle with inflammation. Chronic low-grade inflammation has also been implicated in mediating a wide range of chronic conditions characterized by the presence of fatigue [43]. 

Excessive food consumption leading to obesity may also be a contributing factor to fatigue symptoms. It has been reported that excessive dietary intake, in particular, high fat consumption, may alter sleep parameters (i.e., daytime sleepiness, poor nocturnal sleep quality and sleep apnea), then resulting in fatigue [44,45,46,47]. Moreover, a high-carbohydrate meal (especially in simple sugar) may alter sleep indexes and result in subjective fatigue symptoms [45,48,49]. In fact, in response to ingestion of high-fat and high-sugar foods, circulating levels of glucose, insulin, leptin, cholecystokinin (CCK), peptide YY, and enterostatin are increased, all of which have somnogenic effects [45]. CCK is a short-term satiety hormone released after a fatty meal which could mediate sleep quality parameters and that was also reported to be predictive of and positively correlated with fatigue [49]. Long-term high-fat intake may lead to elevated levels of leptin (an adiposity hormone) and decreased levels of ghrelin [50], which could regulate arousal and wakefulness via orexin [51]. Excessive leptin production causes the release of pro-inflammatory cytokines and has also been reported to contribute to fatigue [52,53,54]. Indeed, unhealthy diet, inflammation and sleep disorders seem to play a pivotal role in the complex etiology of fatigue. 

## 5. Modifications in Body Composition

As mentioned above, changes in food intake during aging may impact nutritional status and eventually contribute to alterations in body composition [55]. In particular, with advancing age, fat mass increases while muscle mass decreases, with a consequent reduction in resting metabolic rate (RMR) [56]. 

### 5.1. Obesity

Despite older people being at a greater risk of undernutrition, in recent years, an increased prevalence of overweight and obesity in those aged 65+ has also been reported [33]. This issue is of particular relevance, as obesity status has consistently been related to increased levels of fatigue [26,57,58,59] (Table 2). In obese people, fatigue may be mediated by systemic inflammation [60]. In fact, individuals with greater adiposity exhibit elevated levels of C-reactive protein, TNF-α, IL-6, leptin and resistin [61,62]. 

Furthermore, obesity status has been related to sleep disorders and vice versa. Sleep disturbances can result in metabolic (decreased glucose tolerance and insulin sensitivity) and endocrine alterations such as decreased levels of leptin, increased evening concentrations of cortisol, increased levels of ghrelin and increased hunger and appetite (all of which promote obesity) [63]. On the other hand, obesity is also characterized by metabolic (i.e., insulin resistance and decreased glucose tolerance) and endocrine (elevated cortisol levels but increased leptin and decreased ghrelin levels) alterations [64,65] in the same domains of sleep disorders. Interestingly, sleep disorders may also be a result of increased levels of pro-inflammatory cytokines [60]. Therefore, obesity could lead to sleep disorders via the elevation of pro-inflammatory cytokines with the consequent onset of fatigue. In fact, obesity appears to be constantly associated to alterations both in qualitative and quantitative sleep domains [58,66]. A number of studies have identified obesity as an important risk factor for obstructive sleep apnea [45,67,68]. Furthermore, daytime sleepiness as well as fatigue is frequently complained of by obese individuals [69]. On the basis of the link among sleep alterations, adiposity and metabolic disorders, obesity could thus represent a ‘chronobiological disease’ [70] mediating fatigue levels. Another interesting issue is that obesity is frequently associated with symptoms that are related to fatigue such as depression [71] and pain [72]. Moreover, excessive body weight, in addition to the hindrance of body mass, may jeopardize the capacity to fulfil many daily tasks, resulting in fatigability.

With aging, there is also a decrease in physical activity levels and an insufficient amount of physical activity has been associated with fatigue [73]. Recent evidence suggests that physical exercise may help reduce fatigue; however, older adults (especially those who are frail and/or obese) with increased levels of fatigue may not have the energy required for exercising and might further decrease their levels of exercise [21,73]. In fact, deconditioned older people frequently show difficulties even in the execution of the simple daily activities.

### 5.2. Undernutrition

Poor nutritional status may be another contributing factor of fatigue. As previously mentioned, fatigue may mimic the exhaustion of the metabolic reserves of the undernourished individual, a kind of alert launched by the organism facing a substantial reduction of its reserves. In fact, fatigue is frequently a symptom of some micronutrient deficiencies [5,74]. What is more, it has been reported that fatigue may also be a result of the dysphagia–malnutrition relationship [75]. Eating an adequate amount of food may, in fact, represent a challenge for dysphagic individuals. The meal is a distressing and fatiguing moment for these persons, who require more time to eat. Additionally, changes in masticatory function (i.e., tooth loss), leading to the selection of soft and easy-to-chew foods due to muscular exhaustion [76], can further negatively affect the nutritional status. In fact, chewing problems and loss of teeth have been associated with both malnutrition [77,78] and fatigue in older people [79]. Dysphagia and masticatory problems could therefore represent disabling conditions fatiguing the aged organism. 

Fatigue might also be looked as a disorder of energy balance since it is frequently reported as a sensation of low energy. Most of the energy consumed during the day (about 60–70%) can be ascribed to the RMR [14]. As mentioned above, aging is accompanied by metabolic derangements, and RMR declines in older people, mainly because of changes in body composition [56]. In addition to these changes, many co-morbidities may further disrupt the biological homeostasis and trigger mechanisms requiring extra energy [80]. For instance, thyroid disorders (which are frequent in older adults) involve derangements in RMR and are often characterized by fatigue [14,81]. In fact, the hypothalamic-pituitary-thyroid axis regulates physiological energy demands (thyroid hormones status correlates with body weight and energy expenditure) [82]. Hyperthyroidism is characterized by a hypermetabolic state associated with increased RMR, weight loss, reduced cholesterol levels, and increased lipolysis and gluconeogenesis [83,84]. On the other hand, hypothyroidism is characterized by hypometabolism with reduced RMR, weight gain, increased cholesterol levels, and reduced lipolysis and gluconeogenesis [85]. These observations could therefore provide a good rationale for a possible correlation between body changes, metabolic alterations and fatigue symptoms. 

### 5.3. Sarcopenia

Sarcopenia is a progressive loss of muscle mass and strength, leading to reduced functional capacity, which is very common in older populations [86]. During aging, there is a net shift toward type I muscle fibers which are slower to contract in comparison with other fibers, but more resistant to fatigue due to a more efficient capacity for ATP production through aerobic metabolism. In parallel, there is both an atrophy and a reduction of motor units in type II fibers which are mainly involved in the muscle loss process. Type II fibers have greater capacity for force production and are faster to contract than type I, but are less energetically efficient and therefore, more subject to fatigue [87,88,89]. Following this rationale, several studies have shown that old individuals develop less muscle fatigue than their younger counterparts during isometric contractions while complaining of greater fatigue during dynamic contractions [90]. However, it is noteworthy that older individuals are more involved in dynamic tasks for ADL, requiring a greater percentage of their reduced maximal strength and then resulting in greater fatigability. Moreover, the majority of the published studies included only healthy individuals and not other different subpopulations (e.g., frail or sarcopenic). 

It is important to bear in mind that sarcopenia is not merely an age-related change of the muscle but a pathological condition defined under specific cut-off measures of muscle mass, strength and function [86]. Indeed, sarcopenia is a multi-factorial process and can include disuse, changes in endocrine function, chronic diseases, inflammation, insulin resistance, mitochondrial abnormalities and nutritional deficiencies [86,91]. Furthermore, muscle fatigue may be defined as the inability of the muscle to produce or maintain force [92]. In fact, the force developed by a skeletal muscle that is sufficient to produce fatigue is a function of the maximum force the muscle can develop. Then, any condition that decreases the maximum muscle force will predispose to fatigue and reduced muscle function [93]. 

It is well known that sarcopenia is characterized by a reduction in not only muscle mass but also muscle strength, leading to decreased functionality. This affects virtually all muscular groups, including respiratory muscles (e.g., diaphragm) [94,95]. In these context, for intermittently contracting muscle like the diaphragm, the critical force to result in fatigue is about 40% of the maximum [96]. A reduction in muscle strength, resulting in a decreased force of contraction of respiratory muscles, can therefore lead to so-called “respiratory fatigue” [93]. Moreover, sarcopenia of respiratory muscles (in particular of the diaphragm), may results in decreased force for expulsive airway clearance tasks (i.e., coughing and sneezing) contributing to increased risk for pneumonia and other respiratory infections [95]. Recent findings have also suggested a relationship between sarcopenia and chronic obstructive pulmonary disease. In these patients, fatigue, together with dyspnea, are the most complained of and distressing symptoms [97]. Respiratory muscle fatigue also may be due to a reduction in energy stores of the muscle [93]. Thus, both the different mechanisms involved in the sarcopenic process and sarcopenia itself, which contribute to an increased susceptibility to stressors, could lead to a decreased muscle ability to resist fatigue. Regarding muscle functionality, the reduction in walking performance and in endurance associated with a perception of exhaustion are all characteristic of sarcopenia [98]. Two of the early symptoms/signs of sarcopenia are, in fact, feeling weak and difficulty rising from a chair, leading to reduced ADL performance [99]. Recently, Patino-Hernandez et al. [100] reported an association between two of the sarcopenia-defining variables (abnormal gait speed and handgrip strength) with fatigue. 

## 6. Mitochondrial Dysfunction and Fatigue

Although there are several mechanisms underlying sarcopenia, mitochondrial dysfunction in skeletal myocytes is considered to be a major factor in age-related muscle decline [102,104]. Mitochondrial damage seems to mark the aging process and vice versa. It is well known that mitochondrial function declines with aging [105] and this might contribute to the onset of age-related conditions [41]. Mitochondria play a pivotal role in bioenergetics, particularly in high energy-demanding tissues like the muscle [106]. Furthermore, mitochondria are a major source of cellular reactive oxygen species (ROS). Under physiologic conditions, ROS mediate cell signaling and survival [107]. However, when in excess, ROS can damage intracellular macromolecules such as proteins, lipids and nucleic acids [102]. Mitochondria are the main source of energy production through the process of oxidative phosphorylation, in which nutrients are harnessed to generate ATP [103,108]. Energy shortage secondary to mitochondrial dysfunction may result in decreased stamina and fatigue [103] (Table 2). Furthermore, inflammation has been linked to mitochondrial dysfunction and oxidative stress [104,109,110]. Pro-inflammatory mediators can, in fact, alter both mitochondrial energy metabolism and function, which, in turn, promote inflammation via oxidant generation and release of damage-associated molecular patterns (DAMPS) [109,110]. Interestingly, fatigue is often observed in many disease conditions characterized by both inflammation and mitochondrial dysfunction [111]. However, given its complexity, the symptom of fatigue cannot be completely explained by a single disease or mechanism. Nevertheless, the central role played by mitochondrial function for many age-related biological pathways may support the identification of the organelles in the generation of the fatigue symptom. In other words, fatigue may become the multidimensional, clinical manifestation of mitochondrial dysfunction. 

Mitochondrial dysfunction can also lead to increased anaerobic metabolism and the production of lactic acid. This is also an interesting concern since acidosis in skeletal muscle can be perceived as muscular fatigue [4]. As a whole, mitochondrial derangements may help better understand the possible link between aging, muscle decline and fatigue, given their role in energy and ROS production [14,102,112].

## 7. Nutritional Interventions

As discussed above, fatigue may be perceived at the muscular level. Thus, nutritional interventions targeted to counteract muscle decline could theoretically also be beneficial in the context of fatigue. Older adults need more proteins to preserve muscle mass compared to younger people [113]. In fact, both the European Society for Clinical Nutrition and Metabolism (ESPEN) [114] and PROT-AGE study group [113] agreed that the current recommended dietary allowance (RDA) for proteins (0.8 g/Kg Body Weight/day) is not sufficient for older persons. Therefore, it has been suggested to increase daily protein assumption at 1–1.2 g/kg body weight/day and up to 1.5 g/Kg/day in the presence of acute or chronic diseases to maintain muscle [113,114]. Furthermore, the per-meal anabolic threshold of protein intake seems to be higher in older individuals (i.e., 25 to 30 g protein/meal, containing about 2.5 to 2.8 g leucine) than young adults [113]. In addition to protein amount, protein source is also important. Plant-based proteins are considered to have a less anabolic effects (partly due to their low content in essential amino acids and leucine) than animal proteins [113,115]. However, it has been suggested that an adequate protein intake can also be achieved by combining plant-based and animal protein sources [115]. Additionally, fast digested proteins seem to better stimulate muscle protein accretion, even if results should be confirmed in larger trials [113]. Some proteins are metabolized to short chain fatty acids (i.e., propionate, butyrate and acetate) which are used by muscle cells to produce energy [116,117,118,119]. Short chain fatty acids also promote muscle anabolism and display anti-inflammatory proprieties [120,121,122,123]. Therefore, it would be interesting to see if protein supplementation may also exert its beneficial effect in alleviating fatigue symptoms. 

However, most evidence for an association between dietary components and fatigue comes from chronic fatigue syndrome (CFS). In particular, some nutritional deficiencies (vitamin C, B vitamins, sodium, magnesium, zinc, folic acid, L-carnitine, L-tryptophan, essential fatty acids, coenzyme Q10) have been reported in CFS subjects [74,124]. Nevertheless, most of the nutritional deficiencies which can contribute to the CFS are frequently observed in older people. Indeed, nutritional treatments targeted to CFS may also have beneficial effects in older adults complaining fatigue. It is important to bear in mind that fatigue, even though is often largely unexplained, may also be the manifestation of an underlying distressing condition. Indeed, each intervention requires a multidimensional approach. Nutritional interventions in older people should be individually adjusted taking into account some components (i.e., nutritional status, physical activity level, disease status) [125]. Some dietary components seem to be promising against the symptom of fatigue. Interestingly, acetyl L-carnitine administration has been reported to reduce both mental and physical fatigue in older people [126] and vitamin D deficiency has been associated with both mental and physical fatigue [101]. Given that fatigue often has been suggested to be due to inflammation and oxidative stress, antioxidant supplementation has been proposed as a strategy to reduce fatigue [43]. Nicotinamide adenine dinucleotide and coenzyme Q10 has been reported to reduce fatigue in CFS patients [127,128]. Additionally, in animal models, it has been documented that antioxidants can lead to a reduction of fatigue [43]. However, the evidence in humans is still sparse [129].

## 8. Conclusions and Future Perspectives

Although fatigue is symptom frequently complained of by older adults, its underlying mechanisms are still incompletely understood. This gap in the knowledge contributes to the poor medical attention to fatigue. Modifications in food intake and changes in body composition, alone or in combination with sleep disorders, seem to influence the perception of fatigue, probably through the mechanisms of inflammation and mitochondrial dysfunction. The history of fatigue may potentially follow what already occurred for the management of pain and depression, conditions similar/related to fatigue which were once neglected but are today major conditions in clinics and research. As occurred for pain and depression, fatigue will obtain the attention it deserves when dedicated research will be developed on this issue, its underlying biological mechanisms will be clarified, possible targets for pharmacological and non-pharmacological interventions will be detected, and awareness will be increased in the medical community.

## Figures and Tables

**Table 1:** Major changes occurring with aging.

Physiological	Pathological	Psychosocial
Digestive system	Diseases	Depression
Hormonal	Medications	Financial status
↓ taste and smell	Neurological disorders	Anxiety
↑ energy expenditure	Swallowing problems	Sleep disorders
Early satiety	Poor dentition	↓ Ability to shop or prepare meals
Cytokines	Poor mobility	Loneliness
Xerostomia		

↓ = decreased, ↑ = increased.

**Table 2:** Overview of discussed studies that explored relationships between fatigue and changes in body composition, inflammation and mitochondrial dysfunction.

References	Study Design and Sample	Aim	Fatigue Assessment	Relevant Results
**Obesity/inflammation**				
Valentine et al., 2009 [61]	Cross-sectional study; 127 community-dwelling older adults.	Assess the contributions of adiposity, systemic inflammation, physical activity/fitness, sleep quality and depression on fatigue.	Two items taken from the Cohen-Hoberman Inventory of Physical Symptoms questionnaire.	Women reported more fatigue than men which was independently associated with inflammation, depression, physical activity and adiposity, whereas in men the only independent predictor was depression.
Valentine et al. 2011 [73]	Cross-sectional study; 182 older people.	Evaluate the influence of weight status, physical activity and inflammation on fatigue.	Multidimensional Fatigue Inventory.	Adiposity independently explained a significant amount of the variance in general and physical fatigue.
Resnick et al., 2006 [26]	Cross-sectional study; 3130 participants aged 20 to 59 years in the NHANES.	Examine the relationships between fatigue and BMI, waist circumference, leisure time physical activity, and macronutrient intake.	Responses to the question, “Right now would you say you are feeling energetic, fresh, average, tired or exhausted?”	Self-reported fatigue was associated with higher BMI, higher waist circumference, and a reduced likelihood of getting recommended levels of physical activity.
Theorell-Haglöw et al. 2006 [59]	Cross-sectional study; 5508 women aged 20 to 60 years.	Analyze the relation between different risk factors and excessive daytime sleepiness and fatigue.	Participants were asked to state how severe their problems were regarding feeling physically tired.	Being overweight was independently related to fatigue and excessive daytime sleepiness.
Lim et al., 2008 [58]	Cross-sectional study; 129 subjects aged 25 to 50 years.	Examine the link between obesity and depressive symptoms.	Short form of Profile of Mood States (POMS–SF) Fatigue/Inertia subscale.	Scores on POMS–SF Fatigue were positively associated with BMI and percent fat.
**Undernutrition/nutritional deficiencies**				
Singh et al., 2014 [38]	Cross-sectional study; 47 nursing home residents.	Examine the correlation between nutritional status and comprehensive physical performance measures.	Functional Ability Questionnaire.	A significant negative correlation was found between self-reported mobility tiredness and BMI.
Westergren et al., 2008 [37]	Cross-sectional pilot study; 89 older people discharged after stroke.	Explore associations between mealtime preparation, eating, fatigue, mood and nutritional status.	Two questions taken from the 12-item Short Form Health Survey.	Having a less favourable nutritional status was significantly predicted by a lack of energy and high age.
Pennisi et al., 2019 [101]	Cross-sectional study; 480 older adults.	Evaluate and compare vitamin D status between older individuals.	Fatigue severity scale (FSS).	Compared with controls, subjects with fatigue showed a significant decrease in vitamin D levels.
**Sarcopenia/mitochondrial dysfunction**				
Christie et al., 2011 [90]	Systematic Review and Meta-Analysis of 37 studies.	Compare the differences in muscle fatigue between young and older adults.	-	Older people develop less muscle fatigue than young adults, particularly during isometric contractions. However, the results also suggest that older adults develop greater fatigue during dynamic contractions, especially when the decline in power is assessed.
Patino-Hernandez et al., 2017 [100]	Cross-sectional study; 1509 older adults.	Examine the association among sarcopenia and its elements with depression and fatigue.	Fatigue was assessed by inquiring the participants: “In the last week: how many times have you felt that everything you do is an effort?”	Sarcopenia did not display statistically significant association with either depression or fatigue. However, both abnormal gait speed and grip strenght (two of the sarcopenia-defining variables) were associated with fatigue.
Wawrzyniak et al., 2016 [102]	48 subjects aged 65+ categorized into idiopathic chronic fatigue (ICF) and non-fatigued (NF) groups	Determine whether skeletal muscle mitochondrial dysregulation and oxidative stress is linked to ICF in older adults.	Functional assessment of chronic illness therapy (FACIT) fatigue scale.	Vastus lateralis muscle biopsies were analyzed, showing reductions in mitochondrial content and suppression of mitochondrial regulatory proteins Sirt3, PGC-1α, NRF-1, and cytochrome C in ICF group compared to NF group.
Filler et al., 2014 [103]	Review; 25 papers of which 20 included patients with CFS/ME, which are summarized here.	Compare associations between fatigue and outcomes of mitochondrial function.	-	Most evidence for lower serum levels of CoQ10 in patients with CFS (4/4 studies). Other findings included reduced carnitine levels (4/5 studies); decreased antioxidant levels (2/2 studies); changes in mitochondrial structure (3/4 studies); and impaired energy production (2/4 studies).
Lacourt et al., 2018 [43]	Review; 46 papers of which 12 included CRF, 20 CFS and 14 animal models.	Compare associations between low-grade inflammation and imbalance in energy availability and expenditure.	-	Most evidence for an association between fatigue and mitochondrial functioning comes from CFS, indicating lower levels of antioxidants and possible reductions in mitochondrial ATP production.

CFS = chronic fatigue syndrome; ME = myalgic encephalomyelitis; CRF = cancer related fatigue; ICF = idiopathic chronic fatigue; NF = non-fatigued; CDC = Centers for Disease Control and Prevention; BMI = body mass index; Sirt3 = sirtuin-3; PGC-1α = Peroxisome proliferator-activated receptor-gamma coactivator-1alpha; NRF-1 = Nuclear respiratory factor 1; CoQ10 = Coenzyme Q10.
